# BubbleGUM: automatic extraction of phenotype molecular signatures and comprehensive visualization of multiple Gene Set Enrichment Analyses

**DOI:** 10.1186/s12864-015-2012-4

**Published:** 2015-10-19

**Authors:** Lionel Spinelli, Sabrina Carpentier, Frédéric Montañana Sanchis, Marc Dalod, Thien-Phong Vu Manh

**Affiliations:** Centre d’Immunologie, de Marseille-Luminy, Aix Marseille University UM2, Inserm, U1104, CNRS UMR7280, F-13288, Marseille, Cedex 09 France; Mi-mAbs (C/O CIML), F-13009, Marseille, France

**Keywords:** Gene set enrichment analysis, Transcriptomic signatures, Comparative transcriptomics, Integrative representation

## Abstract

**Background:**

Recent advances in the analysis of high-throughput expression data have led to the development of tools that scaled-up their focus from single-gene to gene set level. For example, the popular Gene Set Enrichment Analysis (GSEA) algorithm can detect moderate but coordinated expression changes of groups of presumably related genes between pairs of experimental conditions. This considerably improves extraction of information from high-throughput gene expression data. However, although many gene sets covering a large panel of biological fields are available in public databases, the ability to generate home-made gene sets relevant to one’s biological question is crucial but remains a substantial challenge to most biologists lacking statistic or bioinformatic expertise. This is all the more the case when attempting to define a gene set specific of one condition compared to many other ones. Thus, there is a crucial need for an easy-to-use software for generation of relevant home-made gene sets from complex datasets, their use in GSEA, and the correction of the results when applied to multiple comparisons of many experimental conditions.

**Result:**

We developed BubbleGUM (GSEA Unlimited Map), a tool that allows to automatically extract molecular signatures from transcriptomic data and perform exhaustive GSEA with multiple testing correction. One original feature of BubbleGUM notably resides in its capacity to integrate and compare numerous GSEA results into an easy-to-grasp graphical representation. We applied our method to generate transcriptomic fingerprints for murine cell types and to assess their enrichments in human cell types. This analysis allowed us to confirm homologies between mouse and human immunocytes.

**Conclusions:**

BubbleGUM is an open-source software that allows to automatically generate molecular signatures out of complex expression datasets and to assess directly their enrichment by GSEA on independent datasets. Enrichments are displayed in a graphical output that helps interpreting the results. This innovative methodology has recently been used to answer important questions in functional genomics, such as the degree of similarities between microarray datasets from different laboratories or with different experimental models or clinical cohorts. BubbleGUM is executable through an intuitive interface so that both bioinformaticians and biologists can use it. It is available at http://www.ciml.univ-mrs.fr/applications/BubbleGUM/index.html.

**Electronic supplementary material:**

The online version of this article (doi:10.1186/s12864-015-2012-4) contains supplementary material, which is available to authorized users.

## Background

Conventional analyses of high-throughput expression data such as microarray data have long been focusing on the few most regulated genes, with the aim to correlate individual genes with a phenotype of interest. Recent biological discoveries have suggested that many diseases, rather than being the consequence of the strong regulation of a few individual genes, rely on the coordinated regulation of sets of genes contributing to the same biological process [1]. Importantly, moderate but consistent changes in the expression of a significant proportion of genes contributing to a common biological process can lead to a meaningful modification in its activity. Conventional data analyses based on gene-by-gene statistical evaluation of differential expression can often miss this type of informative coordinated regulation of relevant sets of genes, because the fraction of genes individually satisfying the stringent threshold set to define statistically significant changes in expression is too low. Several methods have been designed to solve this issue. One of the most commonly used is the Gene Set Enrichment Analysis (GSEA), which allows statistical assessment of coordinated expression changes of a pre-defined set of genes between pairs of phenotypic conditions [1, 2]. An additional strength of GSEA is that it allows better exploiting the ever increasing knowledge on gene networks and their relationships with biological processes, not only documented contribution to a given biological function as allowed by gene ontology or pathway analyses but also co-expression across a variety of conditions, predicted regulation by a common set of transcription factors, or association with specific diseases as informed by genome wide association studies. Thousands of such gene sets have been carefully curated and regrouped in public databases such as the Molecular Signatures database (MsigDB) [3, 4] or the Stanford Microarray Database (SMD) [5]. Additionally, when comparing results coming from different laboratories or generated on different platforms, the biological and technical variability makes the reproducibility in the regulation of a gene set more robust than in the regulation of a single gene [6–13]. Gene set analyses also allow to better align cell types [14] and physiopathological processes across different species when compared to gene-by-gene analyses, contributing to enable refinement of animal experimentation for advancing the understanding of the cellular and molecular processes at play in human diseases through identification of the model that best represents the targeted human phenotype [15–21]. However, GSEA method is not adapted when one wants to compare gene set enrichment results across multiple pairwise comparisons. Moreover, when GSEA is applied to multiple pairwise comparisons, statistical significance of the enrichments requires to be corrected for multiple testing. Such restriction limits the possibility of interpreting the results in a global context, especially when one deals with many different conditions in a single study, a situation that requires running serially GSEA on all possible pairwise comparisons. Beyond the issues of the time consumed by such large analyses and of the error risk when running many analyses manually, the main difficulty then is to integrate all results together, by inferring “neighbor-to-neighbor” relationships, in order to extract simple and relevant interpretations from the vast amount of enrichments obtained. Moreover, the interpretation of the results being dependent on the biological relevance of the tested gene sets, it is crucial to be able to generate home-made gene sets allowing to rigorously test working hypotheses, for example by being able through the same software suite of extracting relevant, custom gene sets from a first dataset and then of automatically using them for enrichment analysis on a second, independent, dataset. Generation, integrated interpretation and graphical representation in a simple and intuitive way of multiple GSEA is an innovative methodology that has recently been shown to be critical to help rigorously answer important questions in functional genomics. It allowed to better assess the degree of similarities between complex microarray datasets generated in different laboratories or with different experimental models or clinical cohorts [7, 17, 22, 23]. It also facilitated comparison of datasets across microarray platforms, including for identifying homologous cell types across species as we have pioneered [14, 24–28]. However, this approach has been limited so far to only a few research groups having a dual expertise both in a specific area of biology and in bioinformatics. The lack of a computational tool allowing to easily and rigorously perform multiple GSEA without minimal knowledge in bioinformatics and statistics has limited the use of this methodology by biologists. To fill this gap, we have developed and present here a stand-alone program freely downloadable and named Bubble GSEA Unlimited Map or BubbleGUM. It encompasses two modules. The first module, named GeneSign, allows automatically extracting the molecular signature associated to sets of specific biological conditions as compared to one another, consisting in the lists of genes more highly expressed in phenotypes of interest as compared to reference phenotypes, out of a microarray-based expression dataset, using various statistical methods. Examples of molecular signatures are the list of genes which are specifically expressed to higher levels in a given cell type, cell state or disease as compared to many other ones. GeneSign automatically computes molecular signatures and provides the associated heatmaps, through an intuitive easy-to-use interface. The second module allows performing and mining multiple GSEA on all possible pairwise comparisons of an expression dataset. It generates a comprehensive figure called a “BubbleMap”, which provides a transversal comparative point of view of all enrichments, in order to gain insight in the interpretation of multiple inter-connected enrichment plots. Inter-connection of the comparisons is intrinsic to the fact that one deals with multiple conditions that are compared to each other in a pairwise manner. In other words, if gene set X is enriched in condition B when compared to condition A and in condition C when compared to condition B, thus X is enriched in condition C when compared to condition A. Hence, this second module displays these neighbor-to-neighbor relationships through an intuitive schematic representation and therefore allows to better interpret multiple pairwise-limited GSEA results by simplifying their visualization. The two modules can be used either separately or as a suite, since BubbleMap can directly use as gene sets the signatures generated with GeneSign. Finally, BubbleGUM can also be used more broadly to facilitate multi-Omics analyses, since it basically estimates the degree of correlation between lists of molecules associated with intensity signals of any kind, not only including from mRNA hybridization experiments (microarrays) but also from sequencing assays encompassing epigenetic and RNAseq data as well as mass spectrometry data for proteomics, and it should be applicable to metabolomics.

## Methods

### Expression data

All transcriptomic data used in this paper were previously published and were retrieved from public databases, as detailed below. The corresponding experiments were conducted in accordance to ethical rules for experimentation with animals or with human materials, according to the papers where the data were first described [29–31].

### Murine expression data

For mouse immune cell types, our own gene expression dataset was used which included CD8α^+^ cDC (conventional dendritic cells), CD11b^+^ cDC, pDC (plasmacytoïd dendritic cells), B cells, NK (Natural Killer) cells and CD8^+^ T cells, all purified from steady state mouse spleen [29]. The hybridization was performed on Affymetrix mouse 430 2.0 gene chips. Two to three independent replicates were made for each cell type. This dataset was deposited in the Gene Expression Omnibus (GEO) database under reference number GSE9810 [32]. Quality control of the array hybridization was performed through Bioconductor (2.14) [33] in the R statistical environment (version 3.1.0) using the affyPLM package. The raw data was normalized using the RMA (Robust Multichip Average) algorithm using the affy package [34].

### Human expression data

For human immune cell types, a gene expression dataset was compiled from different public sources as previously described [29], in order to include all the cell types known or proposed to be homolog to the mouse cell types under study, namely CD141^+^ cDC, CD1c^+^ cDC, pDC, B cells, NK cells and CD8^+^ T cells, as well as neutrophils as a negative control. These data can be retrieved from ArrayExpress (accession number E-TABM-34 for the DC data) and GEO (accession number GSE72642 for the other cells) [32, 35]. The hybridization was performed on Affymetrix Human Genome U133 Plus 2.0 gene chips. Quality control of the array data was performed through Bioconductor (2.14) in the R statistical environment (version 3.1.0) using the affyPLM package [33]. The raw data was normalized using the RMA algorithm using the oligo package [34].

### Statistical computation of the signature genes by GeneSign

When applicable depending on the method chosen for extraction of the gene signature specific of a phenotype of interest, for each gene present in the expression data file, GeneSign computes a p-value evaluating the risk of being incorrect when declaring that the gene is significantly more highly expressed in the Test population(s) as compared to the Reference population(s). Since GeneSign performs that test over many genes (usually thousands of genes), the probability to declare that a gene is significant, whereas it is not, increases (multiple testing effect).

In order to control this risk, GeneSign applies a multiple testing correction procedure that controls the False Discovery Rate (FDR), i.e. the rate of genes that will be declared significant whereas they are not, compared to the total number of genes declared significant. Specifically, in a first step, the p-values in GeneSign are calculated by computing of a null hypothesis distribution obtained by permutation of the samples. In a second step, these p-values are corrected by using a previously published method [36]. This correction is required because in most cases, the limited number of samples restricts the number of distinct permutations performed, thus leading to the incorrect attribution of 0 values to the estimation of certain p-values. Finally, in a third step, an additional correction is applied, with the Benjamini-Hochberg (B-H) procedure when absence of correlations between values can be assumed, or with the Benjamini-Yekutieli (B-Y) procedure when correlation between values must be assumed [37, 38].

Note that there is one mandatory hypothesis to apply the B-H or the B-Y procedure, which is the uniformity of the p-value distribution under the null hypothesis. We have evaluated the validity of this hypothesis by performing analyses on several cases and several methods used in GeneSign. All those analyses showed that this hypothesis is valid for our methods (data not shown), thus allowing us to apply the B-H and B-Y procedures.

### Correction of multiple testing in the significance of the enrichments in the BubbleMaps

BubbleMap performs several GSEA pairwise comparisons in a row and displays the results as an integrative representation where all the enrichments can be compared to each other. Hence, the risk of false positive detection due to multiple testing effects must be controlled by correcting the p-values of all enrichments across all gene sets and all pairwise comparisons. This type of correction is implemented in GSEA but is limited to the enrichments of gene sets within a pairwise comparison since GSEA performs a single pairwise comparison at a time. In the case of BubbleMap, an additional multiple testing effect occurs, linked to the multiple pairwise comparisons that are performed. To control the FDR on the entire BubbleMap, we apply a Benjamini-Yekutieli (B-Y) procedure to the p-values associated to the normalized enrichment scores (NES) computed by GSEA [38]. These p-values are calculated based on a null hypothesis distribution built from the permutations of either the gene sets or the samples across all the pairwise comparisons performed by BubbleMap. However, since all possible permutations cannot be performed for a matter of computation time, only an estimation of the exact p-value is computed. Like in GeneSign, to better estimate the p-values, BubbleMap applies a correction on the p-value estimations by using a previously published method, before applying the B-Y procedure [36].

## Implementation

BubbleGUM is a stand-alone program developed in Java 1.6. The two modules of BubbleGUM, GeneSign and BubbleMap, can be used either in an independent manner or as a workflow of analyses to assess the enrichment of home-made gene sets and not only of publicly available gene sets. BubbleMap implements the original GSEA algorithm. The file formats used by BubbleGUM and GSEA are fully compatible.

### GeneSign: Generating phenotype signatures from a microarray-based expression dataset

Starting from a pre-processed normalized expression dataset coming from any type of microarray platform, the user can automatically extract the molecular signatures of samples (cell populations, treatments, phenotypes…) of interest as compared to reference samples, using various statistics (see Additional file 1). In this context, a molecular signature is defined as the list of genes that are more highly expressed in the samples of interest (test samples) as compared to the reference samples, according to user’s defined criteria such as the fold change and/or the FDR when applicable (see Additional file 1). GeneSign can extract absolute signatures, using as reference populations all cell samples but the ones for which the signature is computed. Alternatively, GeneSign can extract relative signatures, using as reference populations a set of samples selected by the user. This is interesting when one wants to characterize a population as compared to another one, or when the absolute signature of a population of interest is empty because no gene is specific of the population of interest when compared to all the other populations. In this situation, one can still characterize the population of interest by extracting the genes more highly expressed in that population when compared to a subset of the remaining populations.

GeneSign allows generating transcriptomic signatures by using various statistical methods. The “Min(test) vs Max(ref)” method is applicable to datasets with low replicate numbers, and fast to compute because no permutation procedure is necessary. It is very stringent and yields robust transcriptomic signatures. Therefore, it stands out as a good alternative to permutation-based methods which are less stringent, and which require ideally at least 3 replicates per condition and consume more computing time because they calculate for each gene a p-value and a FDR based on sample permutation. GeneSign proposes several types of permutation-based methods. The first method calculates the ratios of the means: “Mean(test) vs Mean(ref)” method. The second method is more stringent since it calculates the minimal mean ratio among all possible pairwise comparisons of conditions: “Minimal (Pairwise [Mean(test) vs Mean(ref)])” method (see Additional file 1 for an extensive description and comparison of the methods). The third method calculates the signal to noise ratio, defined as the difference of means divided by the sum of the standard deviation of the populations compared: "Signal To Noise". The fourth method computes the minimal signal to noise ratio among all possible pairwise comparisons of conditions: "Minimal pairwise (Signal To Noise)". Results of GeneSign consist in a table with, for each signature gene, the fold change (or signal to noise ratio) calculated according to the method that was chosen, the p-value and FDR if applicable, the number of replicates among the Test populations and among the Reference populations. Each signature that has been generated is displayed in a separate tab. GeneSign also generates a 5-color gene expression heatmap where the genes can be filtered based on their gene symbols or identifiers. The table and the heatmap can be respectively exported as a text file and as a high quality image. Export of the signature gene lists includes the normalized expression data to permit their direct use into third-party programs such as Gene-E or MeV for hierarchical clustering or Principal Component Analysis (PCA) [39]. Alternatively, all signatures can easily be saved into a cart as gene sets and then saved into a proper format to directly assess their enrichment by running classical pairwise GSEA or BubbleMap.

### BubbleMap: Performing multiple GSEA and displaying enrichment patterns

BubbleMap performs GSEA on all possible pairwise comparisons in an expression dataset of interest with the gene sets uploaded by the user, either generated by GeneSign or downloaded from public databases such as MsigDB [3, 4] or SMD [5].

**Fig. 1:**
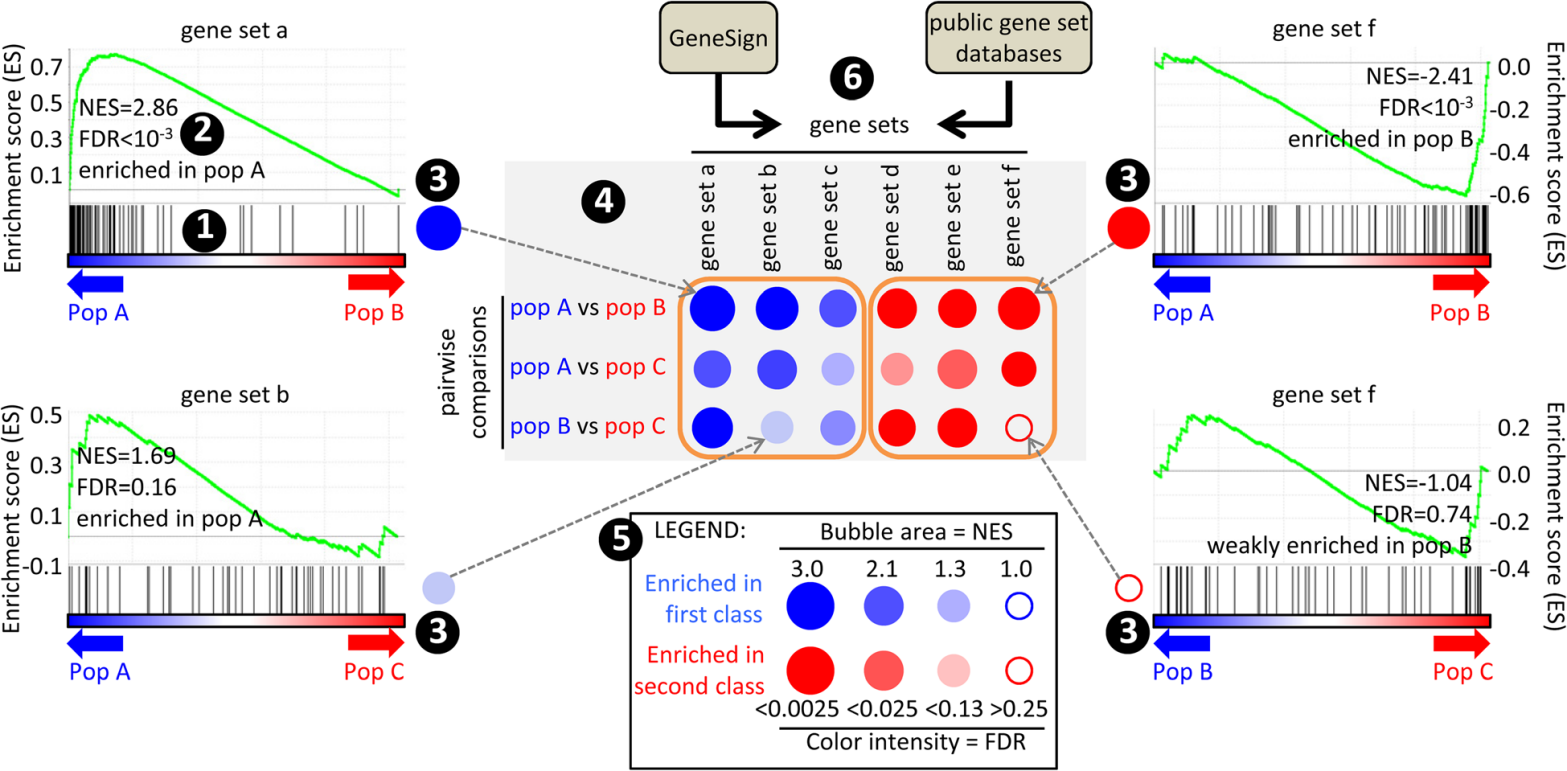
Integrating multiple GSEA into a BubbleMap. GSEA output is a bar code (1) corresponding to the projection of the gene set on the blue-to-red gradient representing all the genes of the chip ranked from high expression in the population on the left to high expression in the population on the right. The more the gene set is regulated, the more the bar code is shifted to one side. This is measured by two parameters. The normalized enrichment score (NES) integrates the number and differential expression intensity of the assessed genes. NES > 0: the gene set is enriched in the population on the left. NES < 0: it is enriched in the population on the right. The FDR is the likelihood that the gene set enrichment represents a false-positive finding (2). Here, the FDR is different from the one calculated by GSEA, because it is corrected for multiple testing across the gene sets and across the pairwise comparisons, thus allowing the user to compare all the bubbles in a BubbleMap. Each enrichment is summarized as a bubble, bigger and darker as the enrichment was stronger and more significant, in a color matching the population in which the gene set was enriched (3). Final output is a BubbleMap (4) with legend (5). Gene sets are either generated by GeneSign or retrieved from public gene set databases (6)

The results are displayed as a figure with colored bubbles of various sizes and color intensities (BubbleMap) (Fig. 1). Each bubble is a GSEA result and summarizes the information from the corresponding enrichment plot. The color of the bubble corresponds to the condition from the pairwise comparison in which the gene set is enriched. The bubble area is proportional to the normalized enrichment score (NES) calculated by GSEA. The intensity of the color corresponds to the statistical significance of the enrichment, calculated through the computation of a permutation-based p-value that is corrected for multiple testing across the various pairwise comparisons. Thus, in addition to the simplicity of performing multiple GSEA in an automatic fashion, BubbleMap optimizes the interpretation of enrichments by allowing the user to compare the results across the pairwise analyses, something which was not possible so far. This is eased by the BubbleMap representation which allows grabbing at a glance on a single computer screen an overview of multiple enrichments across tens of samples and tens of gene sets. The possibility to directly select on the BubbleMap the gene sets and pairwise comparisons of interest, and to reorganize their order, allows to draw focused BubbleMaps for efficient and simple illustration of informative gene set enrichment patterns out of the high amount of information generated by a single analysis. The analyses can be saved as XML files. The BubbleMaps can be exported in high definition images for publication.

## Results and discussion

We illustrate the functioning of BubbleGUM in a workflow successively using GeneSign and BubbleMap to seek for homologies between mouse and human immune cell types based on their sharing of specific molecular signatures/transcriptomic fingerprints [29, 40]. In brief, we use GeneSign to identify the transcriptomic fingerprints of murine immune cell types and we subsequently use these home-made gene sets in BubbleMap to assess their enrichments in human immune cell types (see the online User guide for a detailed step-by-step procedure of how to repeat this data analysis workflow). Cross-species transcriptomic analysis represents a complex challenge due to the fact that the experimental material implies the use of different protocols, platforms, probesets and technologies, therefore leading to the accumulation of multiple sources of variations that are difficult to remove by cross-normalization procedures without adding noise or removing important information. Because it uses the GSEA algorithm, BubbleMap does not require any cross-normalization procedure and is thus very relevant and potent in the context of cross-species transcriptomic comparison.

### Generation of transcriptomic fingerprints specific for murine immune cell types

In the proposed case study, we use a public microarray gene expression dataset for immune cell types purified from mouse spleen and encompassing CD8α^+^ conventional dendritic cells (cDC), CD11b^+^ cDC, plasmacytoid DC (pDC), B cells, NK cells and CD8^+^ T cells [29]. After having normalized this dataset, we used GeneSign to extract the absolute transcriptomic fingerprints of each of the murine immune cell types. We generated 3 different absolute fingerprints for each murine immune cell type: one corresponding to the “Min(test) vs Max(ref) > 1.5x” method, one based on the “Mean(test) vs Mean(ref) > 2x” method with FDR < 0.05 and one based on the “Minimal (Pairwise [Mean(test) vs Mean(ref)]) > 2x” method with FDR < 0.05 using the Benjamini-Yekutieli (B-Y) correction for multiple testing (Additional file 2). It took few seconds to generate all cell-specific transcriptomic fingerprints based on the “Min(test) vs Max(ref)” method, while it took about 2 h to generate all the fingerprints using the “Mean(test) vs Mean(ref)” method based on 1000 permutations, on a personal computer having a 2.4 GHz dual core i7 processor and 8 GB of RAM memory allocated. GeneSign is multi-threaded, the number of cores available impacts on the speed of the analysis. In the murine B cell fingerprints, we found, among the most significant and differentially expressed genes, *CD19*, *CD79A*, *EBF1*, *PAX5* and *FCER2A*, previously reported as involved in the development and functions of B cells. CD19 and CD79A are used as membrane markers to sort this cell population and PAX5 is a master regulator of the B cell lineage differentiation [41]. In the murine CD8α^+^c DC fingerprints, we found, among the most significant, genes involved in the functions of CD8α^+^c DC such as *XCR1*, *TLR3*, *CXCL9* and *CADM1* already reported as being specific of CD8α^+^c DC [29] (Additional file 2). These results thus show that our statistical methods can reliably extract transcriptomic signatures out of an expression dataset.

### Assessing enrichments of the murine immune cell type-specific transcriptomic fingerprints on expression data from human immune cell types

We applied BubbleMap to test the enrichments of the transcriptomic fingerprints of murine immune cell types (Additional file 2) on expression data from human immune cell types encompassing CD8^+^ T cells, B cells, NK cells, neutrophils, CD1c(BDCA1)^+^ cDC, CD141(BDCA3)^+^ cDC and pDC (see Materials and Methods). Since the aim was to test the enrichment of these murine fingerprints on human expression data, the murine fingerprint gene symbols were converted into those of their human orthologs, using the BioMart tool from ENSembl [42]. In order to test the statistical significance of the enrichments obtained with BubbleMap, the GSEA algorithm performs permutations either of the samples or of the gene sets. The human expression dataset is composed of cell types profiled in triplicates, which is not sufficient to perform a sample permutation test as it requires at the very least 5 replicates. Hence, we performed the analysis using the gene set permutation option. The p-values of each enrichment were corrected for multiple testing, to generate FDR values and thus allow for the first time rigorously comparing all the results with each other for many GSEA across multiple pairs of conditions, and hence to allow globally interpreting the BubbleMap (See Materials and Methods). The BubbleMap analysis can be performed with a restricted list of gene sets, as far as most of these genes sets are not expected to be significantly enriched in most GSEA, and over a thousand gene set permutations are performed. However, in order to allow proper functioning of the multiple testing correction procedure, the analysis must include additional gene sets, ideally to be chosen randomly, if a majority of the gene sets is expected to be significantly regulated in most pairwise comparisons. Indeed, the correction for multiple testing is a conservative procedure aiming at decreasing the number of false positives and tending to increase the number of false negatives (see Material and Methods). If it is applied when using only gene sets that are expected to be enriched in all GSEA, this procedure will under-estimate the real number of significantly enriched gene sets. Good sources of additional gene sets can be curated public databases such as MSigDB or SMD [3–5]. In this case study, we added twice the number of the starting gene sets by randomly picking gene sets from collection c3.all.v4.0 from MSigDB. To select all possibly interesting enrichments as a discovery strategy to increase likelihood of discovering novel information, GSEA developers recommend to set the FDR evaluating the statistical significance of the enrichments to a maximal threshold of 0.25 [1]. In our specific study case which is distinct from a discovery strategy but rather a confirmation study where the aim is to identify cell type equivalence relationships that have already been demonstrated in order to validate the relevance of our method, we chose a more stringent 0.10 as the threshold for the FDR. Hence, enrichments with a FDR > 0.10 were considered non-significant and displayed as empty circles. It took about 1 h and 15 min to run the BubbleMap analysis (5000 gene set based permutations) using the murine transcriptomic fingerprints obtained from GeneSign merged together with the randomly picked gene sets (68 gene sets in total), applied to the expression dataset of human immune cell types composed of 21 arrays representing 7 phenotypes (42 pairwise comparisons), on a personal computer with a 2.4 GHz i7 processor and 8 GB of RAM memory allocated (see Additional file 3: Table S1 for an estimation of the computational costs of BubbleMap analyses).

**Fig. 2:**
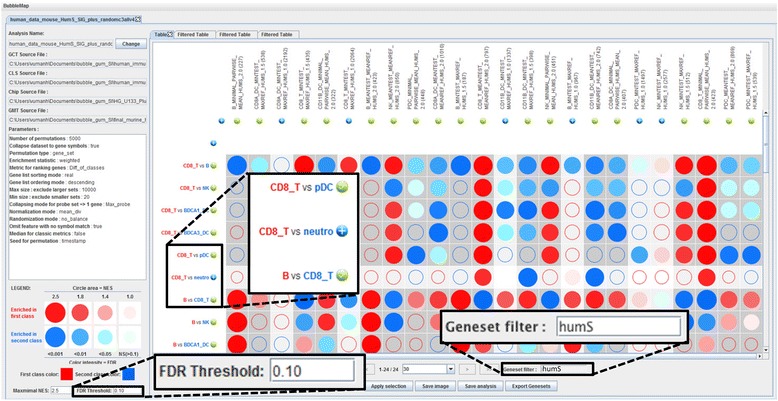
Filtering the gene sets and pairwise comparisons of interest to optimize the BubbleMap: The BubbleMap can be adjusted to focus on significantly enriched (FDR threshold = 0.10) murine signatures. The murine signatures were labeled in GeneSign with “humS” in their names so that they can be retrieved by filtering the entire gene set list by typing “humS” in the dedicated research field. Gene sets and pairwise comparisons of interest are selected by clicking on the + button, then by clicking on “Apply selection”

**Fig. 3:**
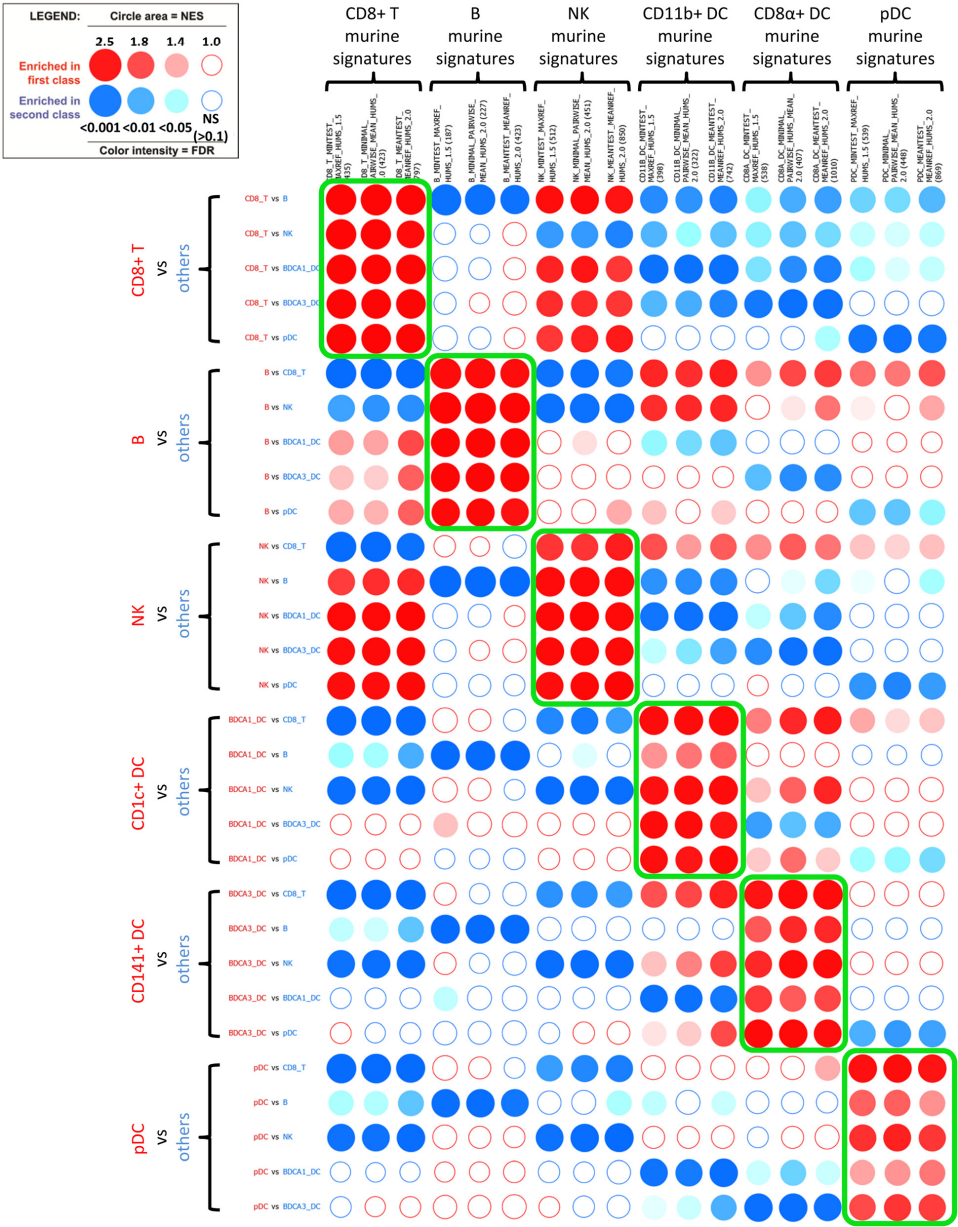
Discovering enrichment patterns from a reorganized BubbleMap. The selection of the gene sets and pairwise comparisons of interest, as well as the re-organization of the gene sets, reveal repetitive bubble enrichment patterns (green boxes)

**Fig. 4:**
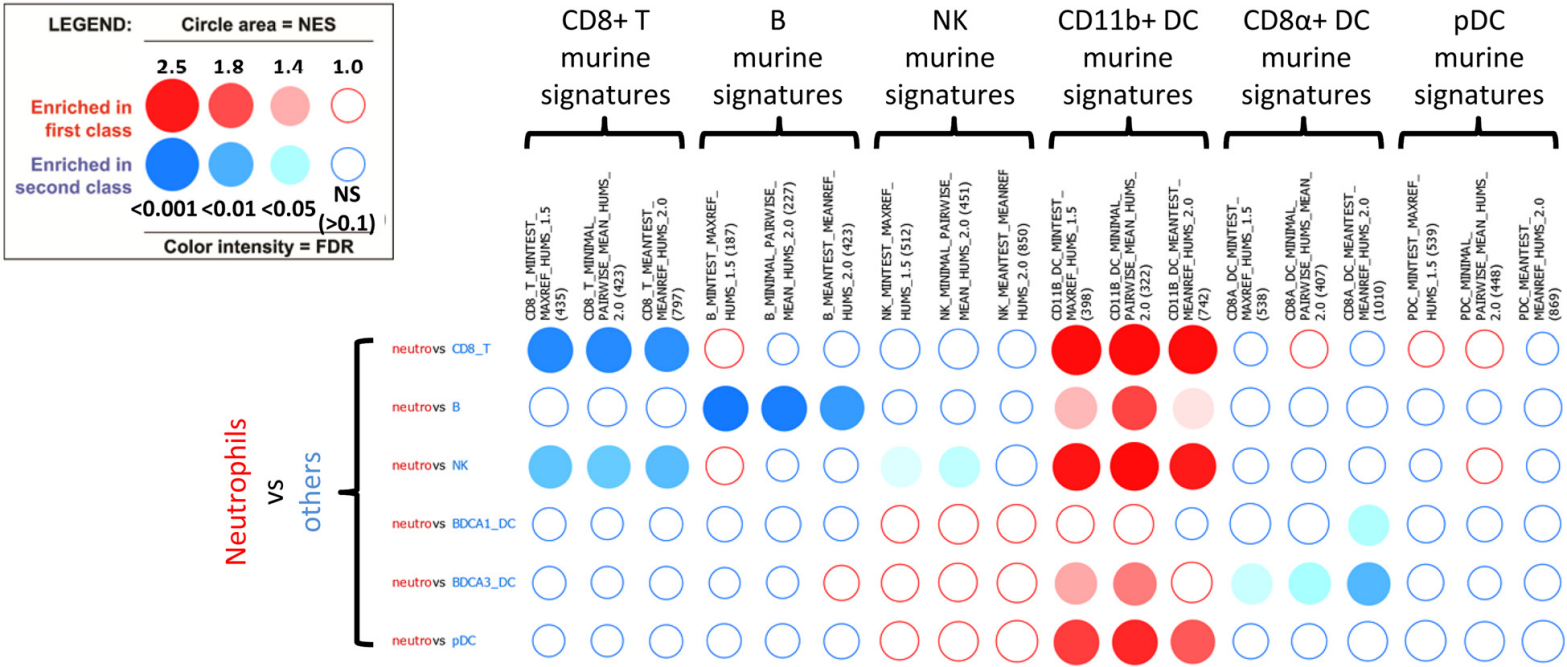
No correlation between the murine cell-specific signatures and the transcriptome of human neutrophils. The BubbleMap of human neutrophils compared to other human immune cell types, using the murine cell-specific signatures, does not reveal any consistent enrichment pattern that is systematically enriched in the neutrophils

Once the analysis has been performed and the results displayed as a BubbleMap, we used the filtering tools for selecting the gene sets and pairwise comparisons of interest. Concretely, out of the entire list of 68 gene sets, we focused on the murine cell-specific transcriptomic fingerprints by typing part of their names (“humS”, for “human symbol”, had been inserted into their names to distinguish the murine fingerprints from the randomly added gene sets) in the dedicated « Geneset filter » field (Fig. 2). Then, we used a simple drag and drop of the gene sets to reorganize the BubbleMap in order to allow easy visualization of enrichment patterns with regards to the hypothesis of conserved expression of molecular signatures between mouse and human immune cell types. Specifically, we grouped together the different fingerprints corresponding to the same murine immune cell type but obtained from GeneSign by using different statistical methods. This selection led to the rapid identification of repetitive patterns of enrichments (Fig. 3), corresponding to the significant enrichments of the cell type-specific murine transcriptomic fingerprints into specific human immune cell types, irrespective of the statistical method used to generate the mouse fingerprints. The murine CD8^+^ T cell fingerprints were found systematically enriched in the human CD8^+^ T cells when compared to any other cell types. Similarly, the murine B cell and NK cell fingerprints were found systematically enriched in the human B cells and NK cells, respectively. As recently demonstrated by our group through a different method [29], the fingerprints of murine CD8α^+^ cDC and CD11b^+^ cDC were found systematically enriched in the human CD141^+^ cDC and CD1c^+^ cDC respectively, and the murine pDC fingerprints were found enriched in the human pDC. In contrast, as a negative control, no remarkable enrichment patterns were observed when examining expression of murine cell type-specific fingerprints on human neutrophils as compared to other human immune cell types (Fig. 4). Thus, human neutrophils were not found to be homologous to any of the mouse immune cell types used in our analyses, in consistency with the lack of neutrophils in the mouse dataset used. However, the murine CD11b^+^ cDC fingerprint was found significantly enriched in human neutrophils as compared to all other human cell populations examined except CD1c^+^ cDC. This enrichment pattern may reflect the strong myeloid signature of mouse CD11b^+^ cDC and human CD1c^+^ cDC, which is well documented and can make difficult the discrimination between these cells and monocyte-derived DC.

Altogether, these results confirm in the case of immune cells the existence of evolutionary conserved molecular signatures specific to cell types [40], and demonstrate how our analysis workflow using BubbleGUM powerfully enables researchers to exploit these signatures for identifying homologous cell types in different species. Moreover, BubbleGUM can also be used as a specific quality control for analysis of expression data from purified cell types, to assess *a posteriori* the risk of cell type cross-contamination and thus, in case of insufficient purity, to prevent erroneous interpretation of the results and to inform the researcher of the necessity to refine the cell sorting strategy.

## Conclusion

The combined use of GeneSign and BubbleMap allowed us in a very simple way to generate transcriptomic fingerprints for murine steady state splenic immune cell types and to assess their enrichments in human blood immune cell types. This analysis showed that the transcriptomic fingerprints for a specific murine immune cell type are systematically enriched in the human immune cell type previously shown/proposed to be its homolog whatever other immune cell type this population was compared to. Hence, we developed a dedicated BubbleGUM bioinformatics tool in order to implement, in a processive, rigorous and easy to interpret way, a strategy for in-depth Omics data mining based on i) transcriptomic signature generation and ii) integration and visual comparison of multiple GSEA. We illustrated the functioning and utility of this strategy and software through their use to confirm homologies between mouse and human immune cell types. In addition, this type of strategy has also been used to identify among several experimental animal models available those that best mimic human pathologies [16–19, 21]. The spectrum of questions to which BubbleGUM can contribute to answer is even considerably larger, since its use can be extended to comparing different types of Omics data, for example to compare mRNA and protein expression in a simple but informative manner, or to examine correlations between mRNA expression and epigenetic modifications at a global scale (unpublished data). Hence, our BubbleGUM software should considerably facilitate integrative analysis of Omics data in many research areas.

## Availability and requirements

Project Name: BubbleGUM

Project home page: http://www.ciml.univ-mrs.fr/applications/BubbleGUM/index.html

Operating system(s): Linux, Mac, Windows

Programming language: Java

Other requirements: Java 7.x (or Java 1.7.x for Linux) or higher (64-bit); a minimum of 4GB of dedicated RAM memory.

License: European Union Public Licence (1.1)

Any restrictions to use by non-academics: written permission from the authors needed.

## Additional files

Additional file 1:
**It provides technical information about the methods used in BubbleGUM and is complementary with the online User Guide (** **http://www.ciml.univ-mrs.fr/applications/BubbleGUM/index.html** **) which is dedicated to provide practical description of the tool.** (PDF 1362 kb) Additional file 2:
**List of the transcriptomic fingerprints for murine splenic immune cell types.** (XLSX 317 kb) Additional file 3: Table S1. Computational cost of BubbleMap. (DOCX 15 kb)

## Abbreviations

GSEA: Gene Set Enrichment Analysis
BubbleGUM: Bubble GSEA Unlimited Map
MSigDB: Molecular Signature Database
SMD: Stanford Microarray Database
cDC: conventional dendritic cells
pDC: plasmacytoïd dendritic cells
NK: Natural Killer cells
FDR: False Discovery Rate
RMA: Robust MultiChip Average
