# Supplementary material for: BubbleGUM: automatic extraction of phenotype molecular signatures and comprehensive visualization of multiple Gene Set Enrichment Analyses
Source: BMC Genomics. 2015 Oct 19;16:814. doi: 10.1186/s12864-015-2012-4 (PMC4617899; doi:10.1186/s12864-015-2012-4)
Supplement: Additional file 1: — It provides technical information about the methods used in BubbleGUM and is complementary with the online User Guide ( http://www.ciml.univ-mrs.fr/applications/BubbleGUM/index.html ) which is dedicated to provide practical description of the tool. (PDF 1362 kb) [file 12864_2015_2012_MOESM1_ESM.pdf]

Bubble GUM: Automatic extraction of phenotype signatures and comprehensive visualization of multiple Gene Set Enrichment Analyses
(Supplementary Information)

Lionel Spinelli[1], Sabrina Carpentier[2], Frédéric Montañana Sanchis[1], Marc Dalod[1] and Thien-Phong Vu Manh[1,§]

[1] Centre d'Immunologie, de Marseille-Luminy, Aix Marseille University UM2, Inserm, U1104, CNRS UMR7280, F-13288 Marseille Cedex 09, France

[2] Mi-mAbs (C/O CIML), F-13009, Marseille, France

§ Corresponding author

This additional file provides technical information about the methods used in Bubble GUM and is complementary with the online User Guide (http://www.ciml.univ-mrs.fr/applications/BubbleGUM/index.html) which is dedicated to provide practical description of the tool.

I.) GeneSign

I.1) The implemented methods to generate transcriptomic signatures

I.2) Statistical calculations of the p-value of the signature genes

I.3) Absolute signatures vs Relative signatures

I.4) Combined signatures

I.5) Description of the GeneSign interface

I.6) Output of GeneSign

I.7) Comparison of the statistical methods

II.) BubbleMap

II.1) Running a BubbleMap analysis

II. 2) Output of BubbleMap

References

I.) GeneSign

I.1) The implemented methods to generate transcriptomic signatures in GeneSign

GeneSign automatically extracts transcriptomic signatures starting from a pre-processed expression dataset. A transcriptomic signature is defined as the list of genes that are more highly expressed in samples of interest (referred to as Test populations) as compared to Reference populations.

Different methods are proposed:

- Min(test) vs Max(ref)

This method calculates the ratio between the lowest replicate value among the Test population(s) (the population(s) for which the signature will be calculated) and the highest replicate value among the Reference population(s). Using this method, the user has to define a minimal fold change (FC) cut-off (Figure 1).

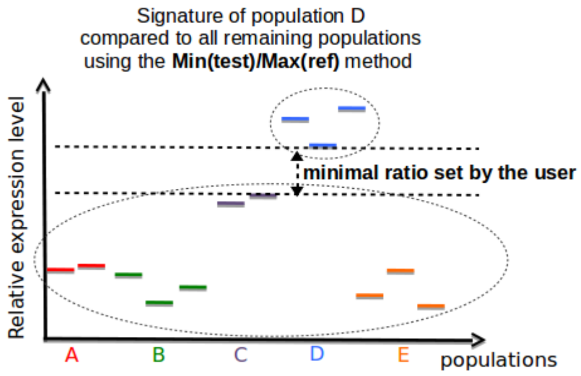

Figure 1: Min(test) vs Max(ref) calculation. Displayed is the relative expression profile of a gene X across 5 different cell populations, A, B, C, D and E. In this example, relative expression values of X in each cell population are either in duplicates or in triplicates.

- Mean(test) vs Mean(ref)

This method calculates the ratio between the mean of the replicate values among the Test population(s) and the mean of the replicate values among the Reference population(s) (Figure 2). With this method, it is highly recommended to consider at least 3 replicates per population. However, conscious that in some situations biological triplicates are difficult to obtain, mean calculations can be performed using duplicates in each population, but results should then be taken cautiously.

Using this method, the user has to set a maximal FDR value (0.05 by default) and can set a minimal fold change (see section I.2 for details about statistics).

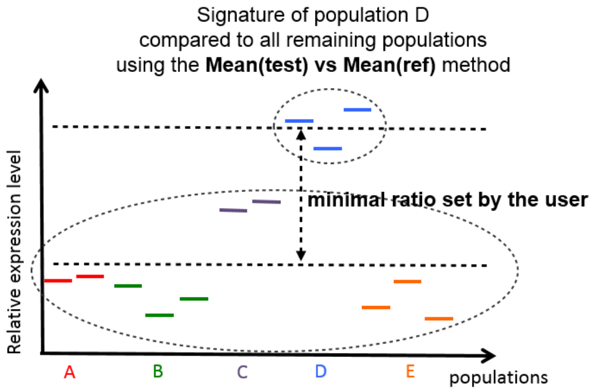

Figure 2: Mean(test) vs Mean(ref) calculation

- Minimal (pairwise [Mean(test) vs Mean(ref)])

This method calculates all possible ratios between the mean of the replicate values among the Test population(s) and the mean of the replicate values for each Reference population and only considers the minimum of these ratios (Figure 3). With this method, it is highly recommended to consider at least 3 replicates per population. However, conscious that in some situations biological triplicates are difficult to obtain, mean calculations can be performed using duplicates in each population, but results should then be taken cautiously.

Using this method, the user has to set a maximal FDR value (0.05 by default) and can set a minimal fold change (see section I.2 for details about statistics).

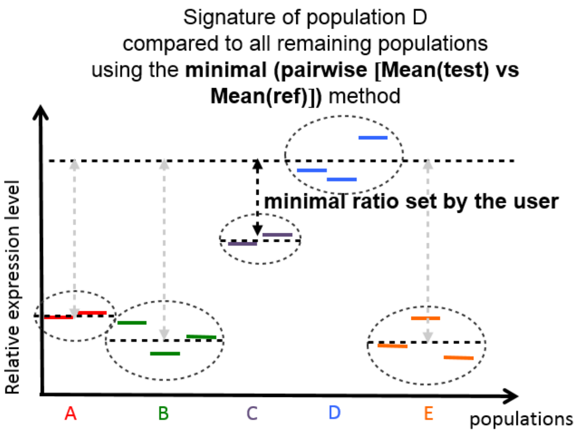

Figure 3: Minimal (pairwise [Mean(test) vs Mean(ref)])

- Signal to noise Ratio

This method computes the Signal To Noise ratio between the replicates among the test population(s) and the replicates among the reference population(s). A minimum of 3 replicates per population is mandatory to use this method.

The Signal to Noise ratio is defined as the difference of means divided by the sum of the standard deviation of the two populations:

$R = (\mu t - \mu r) / (\sigma t + \sigma r)$

where

μt is the mean among the test population

μr is the mean among the reference population

σt is the standard deviation of the test population

σr is the standard deviation of the reference population

Note that is this computation, σ has a minimum value of .2 * absolute(μ), where μ=0 is adjusted to μ=1 (see http://www.broadinstitute.org/gsea/doc/GSEAUserGuideFrame.html section "Metric for gene ranking"). The larger the value of this ratio, the more different the gene expression in each geneset.

Using this method, the user has to set a maximal FDR value (0.05 by default) (see figure 9 and "Understanding statistical

computation" below).

- Minimal (pairwise [Signal to noise Ratio])

This method computes all possible Signal To Noise ratios between the replicates among the test population(s) and the replicates for each population of the reference populations and considers the minimum of these ratios (see definition of Signal to Noise ratio above). A minimum of 3 replicates per population is mandatory to use this method.

Using this method, the user has to set a maximal FDR value (0.05 by default) (see figure 9 and "Understanding statistical computation" below).

I.2) Statistical calculation of the p-value of the signature genes

GeneSign proposes to compute a p-value for each gene of the expression dataset, by performing permutations of the samples in order to extract the genes that are significantly more expressed in the Test population as compared to the Reference population, based on a minimal fold change (FC) and/or a maximal False Discovery Rate (FDR) set by the user.

The computation of the p-values, i.e the probability to declare that a gene is significant whereas there is no difference in the expression, is made as follows:

- For each gene, the statistical value (fold change value (FC) or Signal to Noise ratio) between the Test population(s) and the Reference population(s) is computed, according to the method chosen by the user.
- Statistical significance is assessed by performing permutations. Permutations are performed by mixing the population replicates. In the specific case of the "Minimal (pairwise [Mean(test) vs Mean(ref)])" or the "Minimal (pairwise [Signal To Noise])"methods, the number of replicates per population is conserved to remain similar to the initial distribution. Moreover, permutations are done in such a way that they do not reproduce the conditions of the initial distribution. The number of permutations can be controlled by the user through the software preferences, but cannot be set to less than 1000.
- Each permutation produces, for each gene, a statistical value (fold change or signal to noise ratio) between the Test population(s) and the Reference population(s), according to the method chosen by the user.

- The p-value for each gene is computed as the ratio of statistical values obtained by permutations for this gene, that are greater than the actual gene statistical value, divided by the number of computed permutations. In most cases, the limited number of samples will permit to perform only a limited number of different permutations, inducing the computation p-values equal to 0 which have no true meaning. This imprecision is corrected according to the procedure proposed by Phipson et al. However, it has the limitation that the best p-values cannot be smaller than a value depending on the number of permutations (for instance, it will be 9.99e-4 for 1000 permutations).

Since GeneSign performs these p-value computations over several genes (usually thousands of genes), the risk of false discovery increases (multiple test effect). In order to control the error due to multiple testing, a FDR control procedure is proposed by applying the Benjamini-Hochberg (B-H) procedure (when absence of correlations between values can be supposed) or the Benjamini-Yekutieli (B-Y) procedure (when correlation between values must be assumed) (Hochberg et al., 1990; Reiner et al., 2003). The user is invited to enter a FDR threshold (0.05 by default) and the resulting signature will only contain the genes for which the p-value will ensure the final FDR will be lower than the threshold set by the user.

Precision and validity of those computations are based on the number of permutations and the number of replicates in the populations. Those statistical computations are not made if the number of replicates for the Test or Reference populations is too low (signatures are produced but without p-values and FDR control). Moreover, increasing the number of permutations could help improving the precision of the p-value computations and therefore the result of the B-H or B-Y procedures.

Important note: It could happen that the result of a GeneSign analysis may identify no significant signature gene (empty signature) whereas one could expect the signature not to be empty, using as parameter a certain minimal fold change. This absence of significant signature gene could be the consequence of the multiple test correction associated with the lack of precision in the calculation of the lowest p-values, due to the limitation of sample permutations. Indeed, the stringency of the correction increases with the number of genes that are tested, and could exceed the level of p-value precision (which is limited by the number of permutations performed). In that case, the number of genes that are still significant after the correction is null and the returned signature is empty. To avoid this limitation, one may want to use a greater fold change threshold. Increasing

this threshold will reduce the number of initial genes that will be tested for multiple test correction, implying a less stringent correction on more significant p-values, thus bringing results with a lower false negative ratio.

I.3) Absolute signatures vs Relative signatures

GeneSign provides the possibility to generate absolute and relative transcriptomic signatures.

An absolute signature is defined as the list of genes that are more highly expressed in the Test population(s) as compared to all the remaining populations that are present in the expression dataset (Figure 4A).

A relative signature is defined as the list of genes that are more highly expressed in the "Test population(s)" as compared to other populations that have been chosen as Reference by the user (Figure 4B).

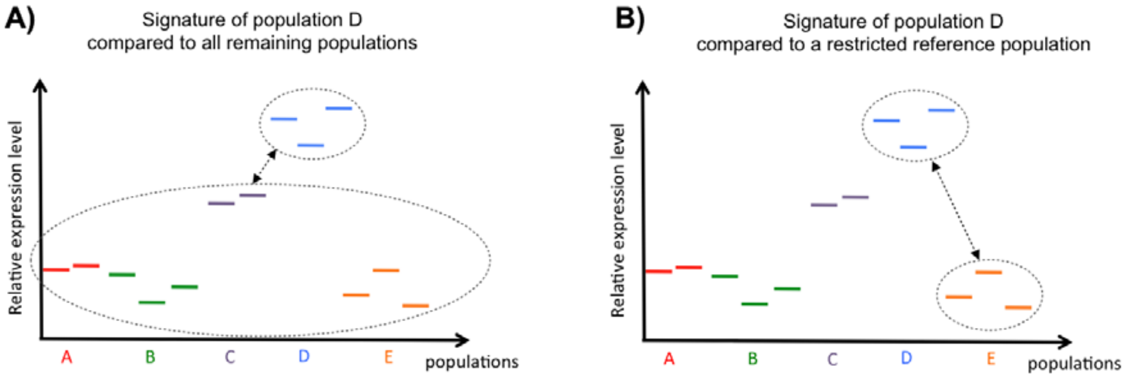

Figure 4: Absolute vs Relative signatures. GeneSign provides the option to generate "absolute" (A) and "relative" (B) transcriptomic signatures.

I.4) Combined signatures

On top of the fact that GeneSign provides the possibility to generate both absolute and relative transcriptomic signatures, it also provides the option to generate combined transcriptomic signatures, based on the merging of various Test populations. Here again, combined transcriptomic signatures can be generated using all the remaining populations as Reference (absolute) (Figure 5A) or using a restricted Reference population (relative) (Figure 5B).

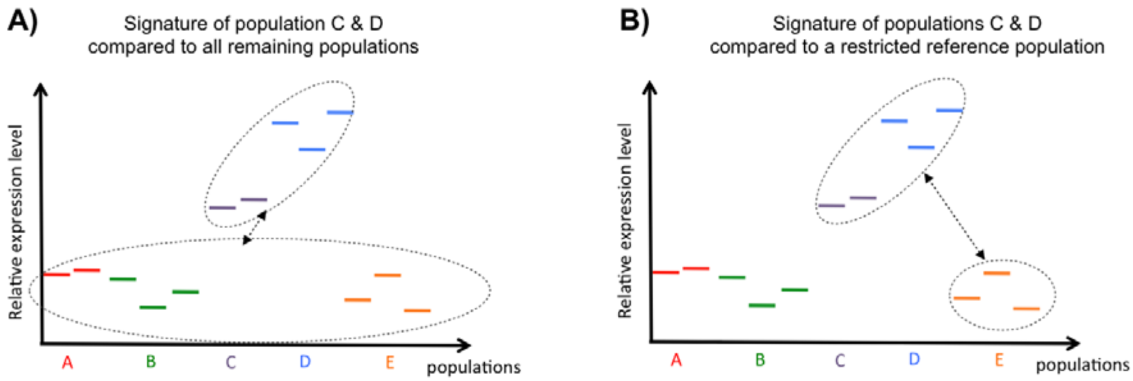

Figure 5: Combined signatures. GeneSign provides the option to generate combined transcriptomic signatures, either as absolute (A) or relative (B) transcriptomic signatures.

I.5) Description of GeneSign interface

GeneSign interface is intuitive (Figure 6). The software only requires two files, a normalized expression datafile (.gct) and a class file (.cls).

See the following links for the description of the respective file formats:

http://www.broadinstitute.org/cancer/software/gsea/wiki/index.php/Data_formats#GCT:_Gene_Cluster_Text_file_format_.28.2A.gct.29

http://www.broadinstitute.org/cancer/software/gsea/wiki/index.php/Data_formats#CLS:_Categorical_.28e.g_tumor_vs_normal.29_class_file_format_.28.2A.cls.29

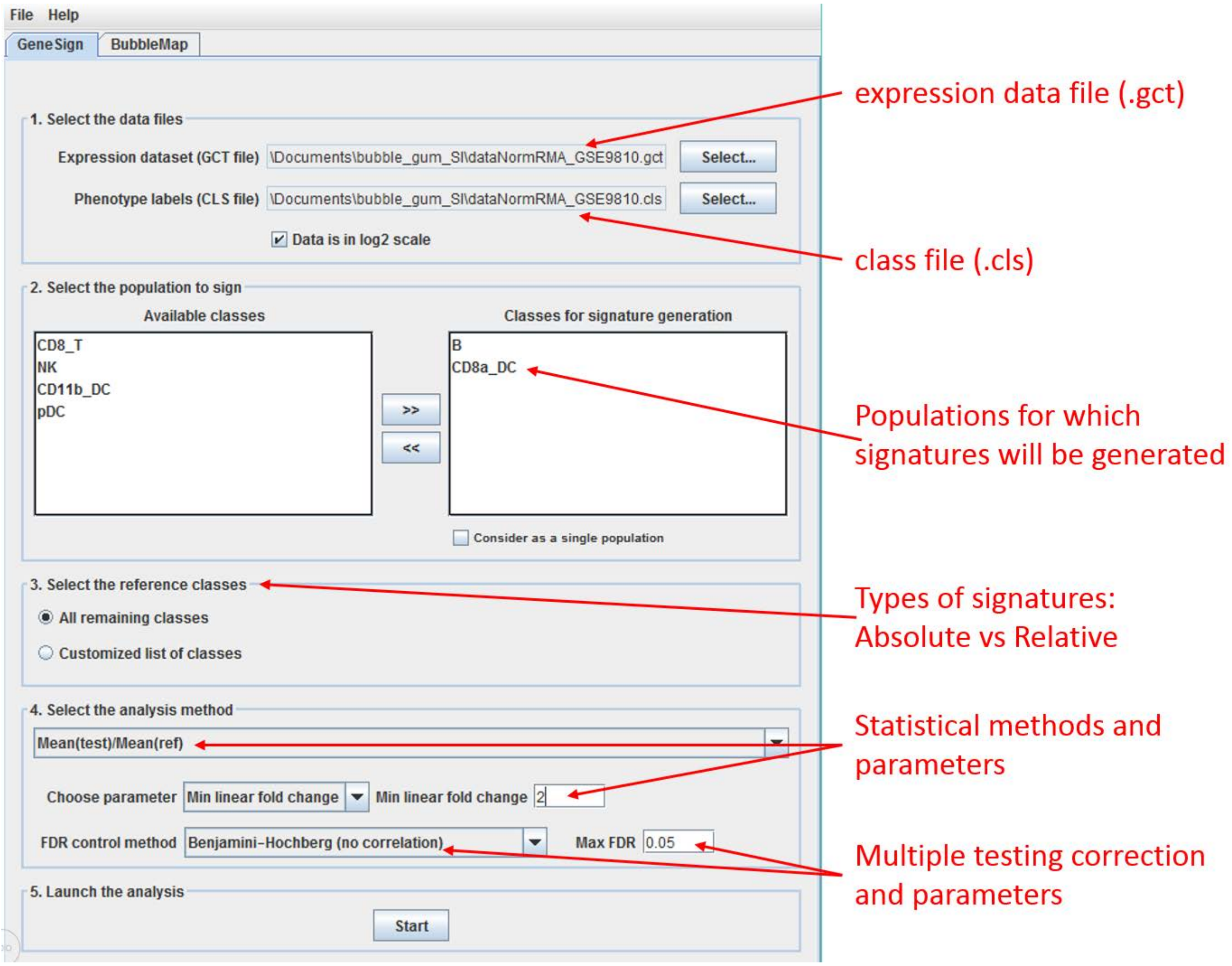

Figure 6: Interface of GeneSign. An easy-to-use interface allows the user to select the signatures to be generated and the statistical method to compute these signatures.

I.6) Output of GeneSign

GeneSign provides two kinds of output:

- First, GeneSign opens a table displaying the signature genes and the fold changes calculated according to the method that was chosen, the p-value if applicable, the number of replicates among the Test populations and the number of replicates among the Reference populations (Figure 7). Each signature that has been generated is displayed in a separate tab. Each signature (with corresponding expression values across all samples) can be exported into a text file, in order to be uploaded into other programs.

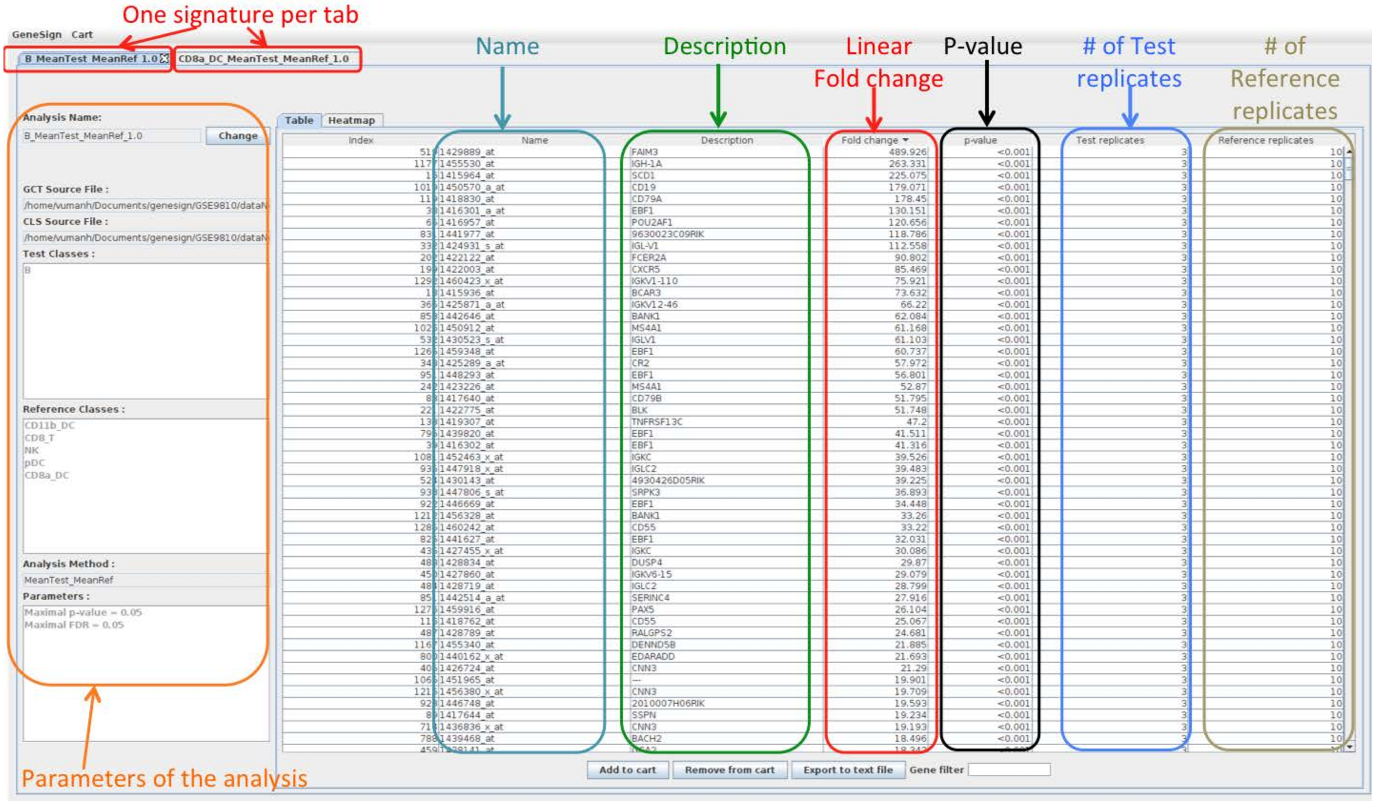

Figure 7: Table displaying the signature genes. The left panel displays the parameters of the analysis. The right panel displays the table, including the gene identifier, gene name, the fold change between the Test and Reference populations, the p-value when applicable, the number of samples in the Test population(s) and the number of samples in the Reference population(s).

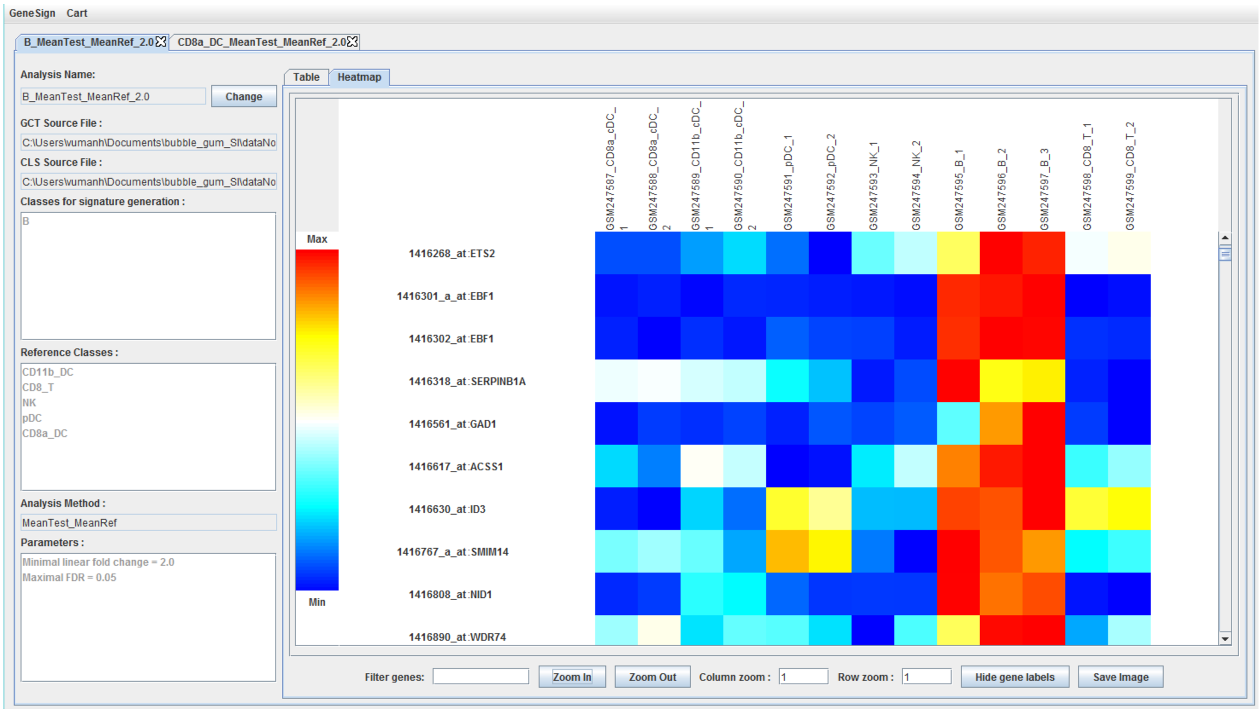

Figure 8: Heatmap representing the relative expression profile of the signature genes across all conditions present in the expression dataset. Each line corresponds to a signature gene and each column represents a sample.

- Second, GeneSign also generates a 5-color heatmap of the signature genes (Figure 8). In this heatmap, the gene labels can be hidden to obtain a more compact view. A zoom in/out button, as well as Column/Row zoom options allow changing the display of the heatmap. The heatmap can be saved as a high-definition (svg) or rastered (png) image.

In both the table and the heatmap, one can filter the genes by typing the gene name or part of the gene name in the « Filter gene » field (Figure 9).

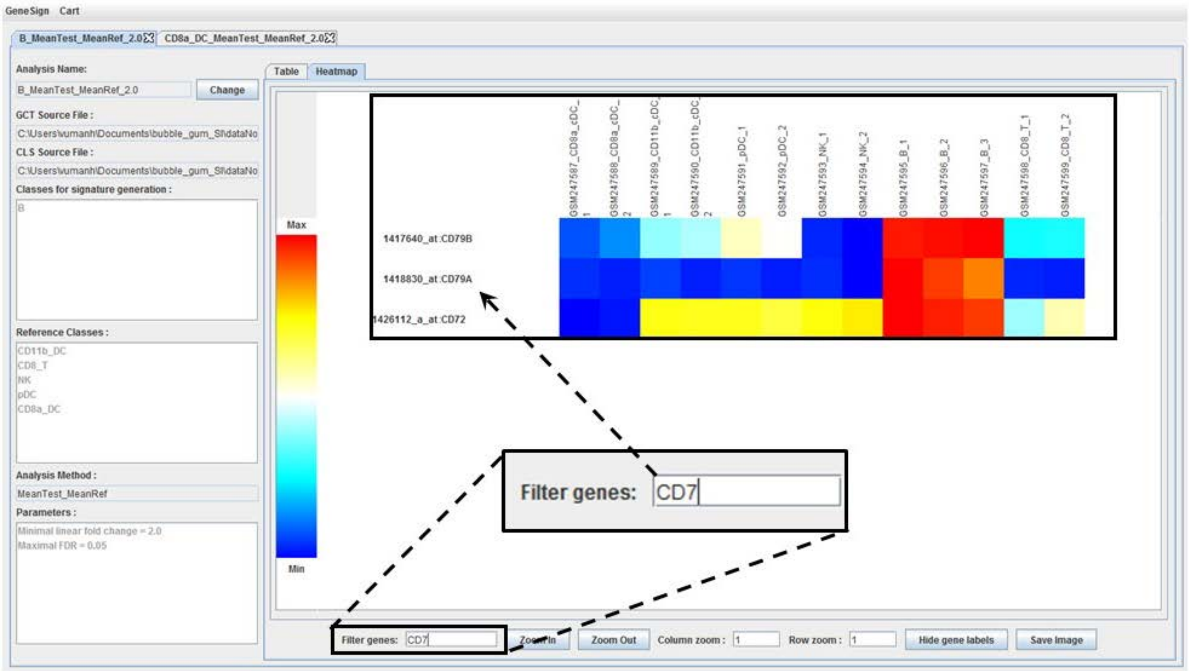

Figure 9: Filtering of the genes of interest. It can be applied on both the table and the heatmap.

When the transcriptomic signatures of interest have been generated, they can be added to a cart (i.e. a temporary file). The cart keeps in memory the transcriptomic signatures as long as the program is opened, and it can be completed with new/tuned signatures. Once the definitive transcriptomic signatures have been obtained and added to the cart, the content of the cart can then be exported into a gene set file (.gmt) that is compatible with BubbleMap and GSEA (Subramanian et al. 2005), in order to assess the enrichment of these signatures on a third-party dataset.

I.7) Comparison of the statistical methods

In order to compare the methods of signature generation that are implemented in GeneSign, we have extracted, based on the public murine immune cell dataset used in the analysis workflow (See materials and methods), the transcriptomic signatures of the B cells, the CD8+ T cells, the CD8〈+ and CD11b+ cDC, the NK cells and of the pDC, according to 3 (or 4 when the number of replicates is sufficient) different methods and parameters:

1) Min(test) vs Max(ref) with FC > 1.5x

2) Mean(test) vs Max(ref) with FC > 2x and FDR < 0.05

3) Minimal (Pairwise [Mean(test) vs Mean(ref)]) with FC > 2x and FDR < 0.05

4) Signal To Noise Ratio with FDR<0.05

(FC : Fold Change)

The lists of signature genes, for each method and for each murine immunocyte, are available as additional file 1.

The summary of the different signatures we have extracted, with the corresponding number of unique genes, is provided as Table 1.

Test population	Reference population	Method	min Fold change	max FDR	size (# genes)
B	all remaining	Min(test) vs Max(ref)	1.5	NA	187
B	all remaining	Mean(test) vs Mean(ref)	2	0.05	423
B	all remaining	Minimal (Pairwise [Mean(test) vs Mean(ref)])	2	0.05	225
B	all remaining	Signal To Noise Ratio	NA	0.05	1107
CD8+ T	all remaining	Min(test) vs Max(ref)	1.5	NA	435
CD8+ T	all remaining	Mean(test) vs Mean(ref)	2	0.05	797
CD8+ T	all remaining	Minimal (Pairwise [Mean(test) vs Mean(ref)])	2	0.05	423
CD8+ T	all remaining	Signal To Noise Ratio	NA	0.05	NA
CD8+ DC	all remaining	Min(test) vs Max(ref)	1.5	NA	538
CD8+ DC	all remaining	Mean(test) vs Mean(ref)	2	0.05	1010
CD8+ DC	all remaining	Minimal (Pairwise [Mean(test) vs Mean(ref)])	2	0.05	407
CD8+ DC	all remaining	Signal To Noise Ratio	NA	0.05	NA
CD11b+ DC	all remaining	Min(test) vs Max(ref)	1.5	NA	398
CD11b+ DC	all remaining	Mean(test) vs Me(an(ref)	2	0.05	742
CD11b+ DC	all remaining	Minimal (Pairwise [Mean(test) vs Mean(ref)])	2	0.05	322
CD11b+ DC	all remaining	Signal To Noise Ratio	NA	0.05	NA
NK	all remaining	Min(test) vs Max(ref)	1.5	NA	512
NK	all remaining	Mean(test) vs Mean(ref)	2	0.05	850
NK	all remaining	Minimal (Pairwise [Mean(test) vs Mean(ref)])	2	0.05	451
NK	all remaining	Signal To Noise Ratio	NA	0.05	NA
pDC	all remaining	Min(test) vs Max(ref)	1.5	NA	539
pDC	all remaining	Mean(test) vs Mean(ref)	2	0.05	869
pDC	all remaining	Minimal (Pairwise [Mean(test) vs Mean(ref)])	2	0.05	448
pDC	all remaining	Signal To Noise Ratio	NA	0.05	NA

Table 1: list of murine transcriptomic signatures that we have generated, and subsequently used in BubbleMap.
NA: not applicable; min, minimal; max, maximal

As a general observation, we observe that the Mean(test) vs Mean(ref) with FC>2x and FDR<0.05 method is the one which gives the largest signatures, whatever cell type considered.

The Min(test) vs Max(ref)>1.5x method and the Minimal (Pairwise Mean(test) vs Mean(ref)) with FC>2x and FDR<0.05 return

cell specific signatures which are more or less similar in terms of size.

In order to better evaluate the relative stringency of the statistical methods implemented in GeneSign, we compared the different cell specific signatures obtained by the different methods and parameters listed in Table 1, by analyzing their overlaps using Venn diagram representations with JVenn (Bardou et al. 2014).

- Comparison of the B cell specific signatures

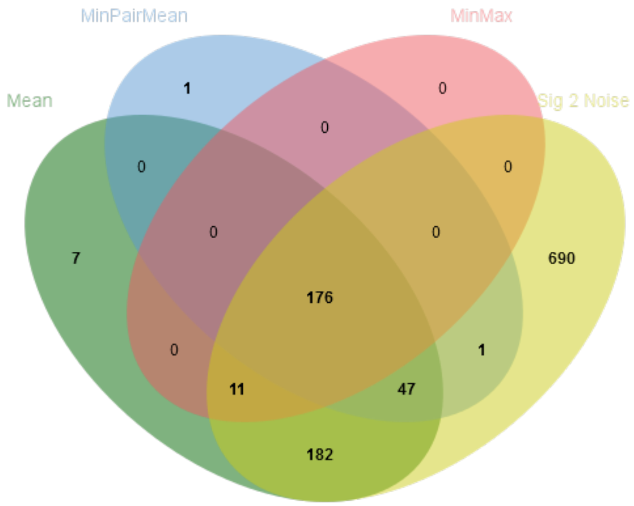

Figure 10: Venn diagram of the B cell signatures obtained by 4 different methods: Mean(test) vs Mean(ref)>2x (green), Minimal (Pairwise [Mean(test) vs Mean(ref)])>2x (blue), Min(test) vs Max(ref)>2x (red) and Signal to Noise ratio (FDR<0.05).

- Comparison of the CD8+ T cell specific signatures

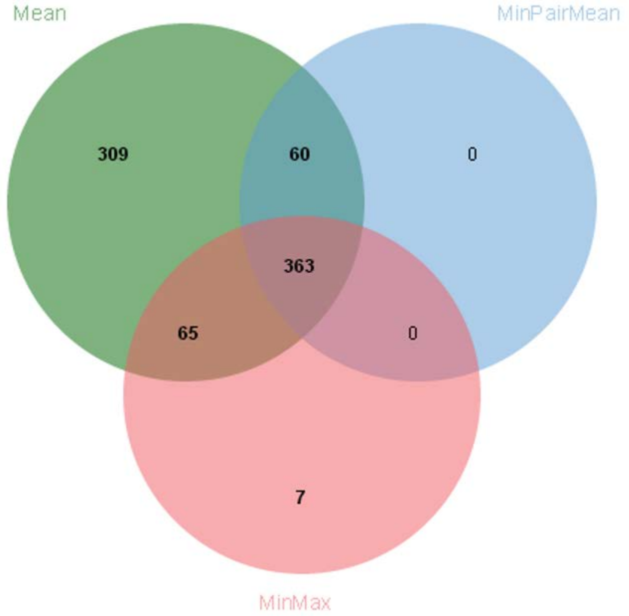

Figure 11: Venn diagram of the CD8+ T cell signatures obtained by 3 different methods: Mean(test) vs Mean(ref)>2x (green), Minimal (Pairwise [Mean(test) vs Mean(ref)])>2x (blue) and Min(test) vs Max(ref)>2x (red).

- Comparison of the CD8+ cDC specific signatures

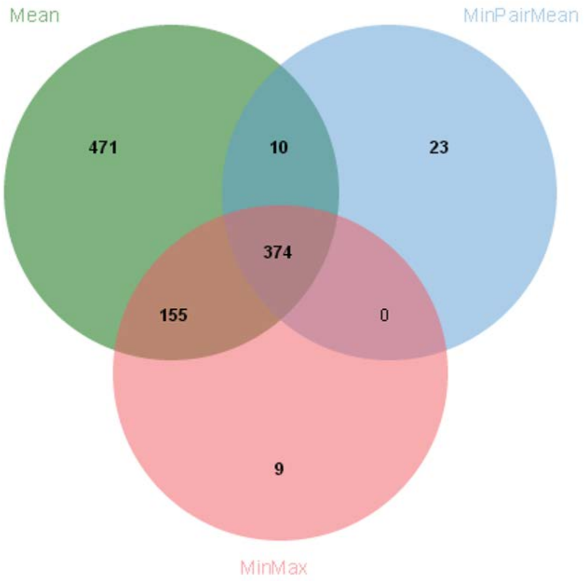

Figure 12: Venn diagram of the CD8α+ DC signatures obtained by 3 different methods: Mean(test) vs Mean(ref)>2x (green), Minimal (Pairwise [Mean(test) vs Mean(ref)])>2x (blue) and Min(test) vs Max(ref)>2x (red).

- Comparison of the CD11b+ cDC specific signatures

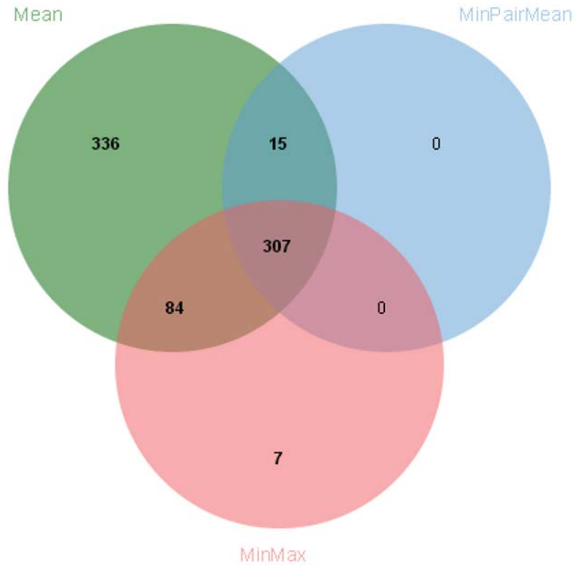

Figure 13: Venn diagram of the CD11b+ DC signatures obtained by 3 different methods: Mean(test) vs Mean(ref)>2x (green), Minimal (Pairwise [Mean(test) vs Mean(ref)])>2x (blue) and Min(test) vs Max(ref)>2x (red).

- Comparison of the NK cell specific signatures

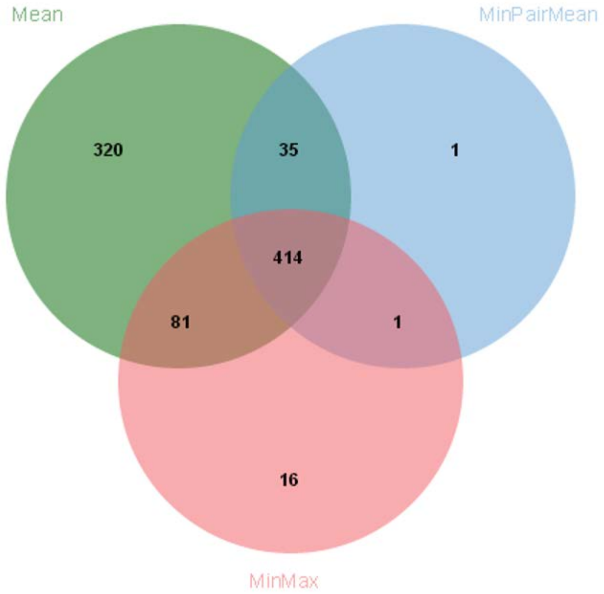

Figure 14: Venn diagram of the NK cell signatures obtained by 3 different methods: Mean(test) vs Mean(ref)>2x (green), Minimal (Pairwise [Mean(test) vs Mean(ref)])>2x (blue) and Min(test) vs Max(ref)>2x (red).

- Comparison of the pDC specific signatures

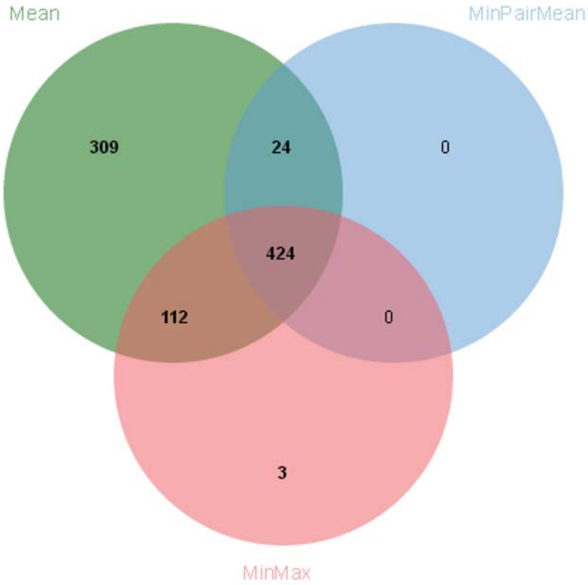

Figure 15: Venn diagram of the pDC signatures obtained by 3 different methods: Mean(test) vs Mean(ref)>2x (green), Minimal (Pairwise [Mean(test) vs Mean(ref)])>2x (blue) and Min(test) vs Max(ref)>2x (red).

In general, except for few exceptions, we observe that for each cell type considered, the genes that were found as signature genes in the Min(test) vs Max(ref)>1.5x method and in the Minimal (Pairwise [Mean(test) vs Mean(ref)]) with FC>2x and FDR<0.05 were also found as signature genes by the Mean(test) vs Mean(ref)>2x with FDR<0.05 method which extracted much more genes than the two other methods. However, the Min(test) vs Max(ref)>1.5x method and the Minimal (Pairwise [Mean(test) vs Mean(ref)]) with FC>2x and FDR<0.05 displayed significant differences in their lists of signature genes although a vast majority of their signature genes was shared between the two methods.

Overall, these results thus suggest that, at least using this murine expression dataset, the Min(test) vs Max(ref)>1.5x method and the Minimal (Pairwise [Mean(test) vs Mean(ref)]) with FC>2x and FDR<0.05 method are more stringent than the Mean(test) vs Mean(ref)>2x with FDR<0.05 method. This also shows that the Min(test) vs Max(ref) method represents a good alternative to permutation-based methods in the purpose of transcriptomic signature generation.

II.) BubbleMap

II.1) Running a BubbleMap analysis

Because it uses the original GSEA algorithm, BubbleMap has been developed in such a way that users of GSEA will feel comfortable when using BubbleMap: The BubbleMap interface is very similar to the GSEA java interface.

Like GSEA, BubbleMap requires four different files to perform an analysis:

- a normalized expression dataset (.gct)

- a class file (.cls)

- a chip file containing the annotations of the chip (.chip)

- a gene set file (.gmt or .gmx), coming from GeneSign or from a public gene set database

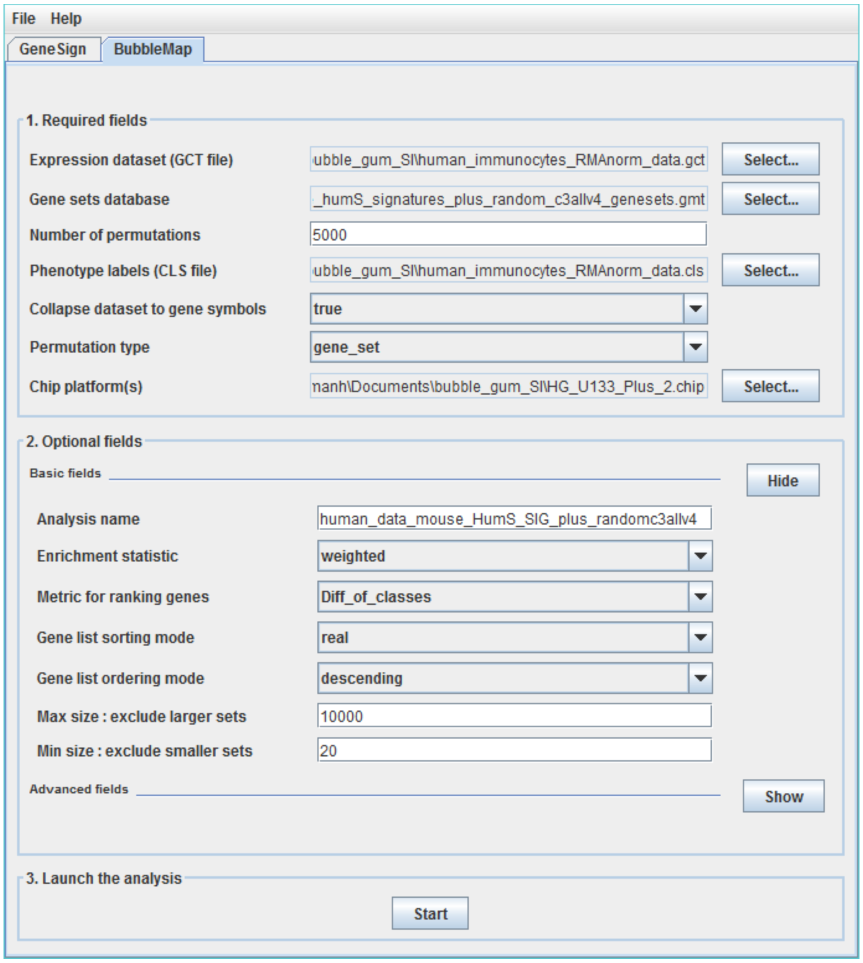

Figure 16: BubbleMap interface. It is similar to the GSEA java interface. Description of these parameters is available in the User Guide.

A detailed description of the four files is provided in the online User guide and here:

http://www.broadinstitute.org/cancer/software/gsea/wiki/index.php/Data_formats

The files used in the analysis workflow described in the manuscript are temporarily available to the reviewers on the Bubble GUM web server: http://www.ciml.univ-mrs.fr/applications/BubbleGUM/index.html

Once the 4 files have been uploaded, the user has to select the parameters of the analysis. A detailed description of the parameters is provided in the Bubble GUM online User's guide and also here:

http://www.broadinstitute.org/gsea/doc/desktop_tutorial.jsp

We recommand the user to carefully read the description of the parameters before launching an analysis.

The parameters that were used in the analysis workflow described in the manuscript are shown in Figure 16.

II.2) Output of BubbleMap

The results of BubbleMap are displayed as a figure with colored bubbles of various sizes and color intensities (bubble map). Each bubble is a GSEA result and reflects a GSEA enrichment plot. Each line represents a pairwise comparison and each column represents a gene set. The color of the bubble corresponds to the condition from the pairwise comparison in which the asse ssed gene set is enriched, the bubble area is proportional to the normalized enrichment score (NES) calculated by GSEA, and the intensity of the color corresponds to the p-value calculated by GSEA and corrected for multiple testing by BubbleMap so that all the bubbles can be compared to each other, across the gene sets and across the pairwise comparisons (Figure 17). A non-significant enrichment is displayed as an empty bubble (the "non-significant" FDR threshold can be set by the user in the legend panel).

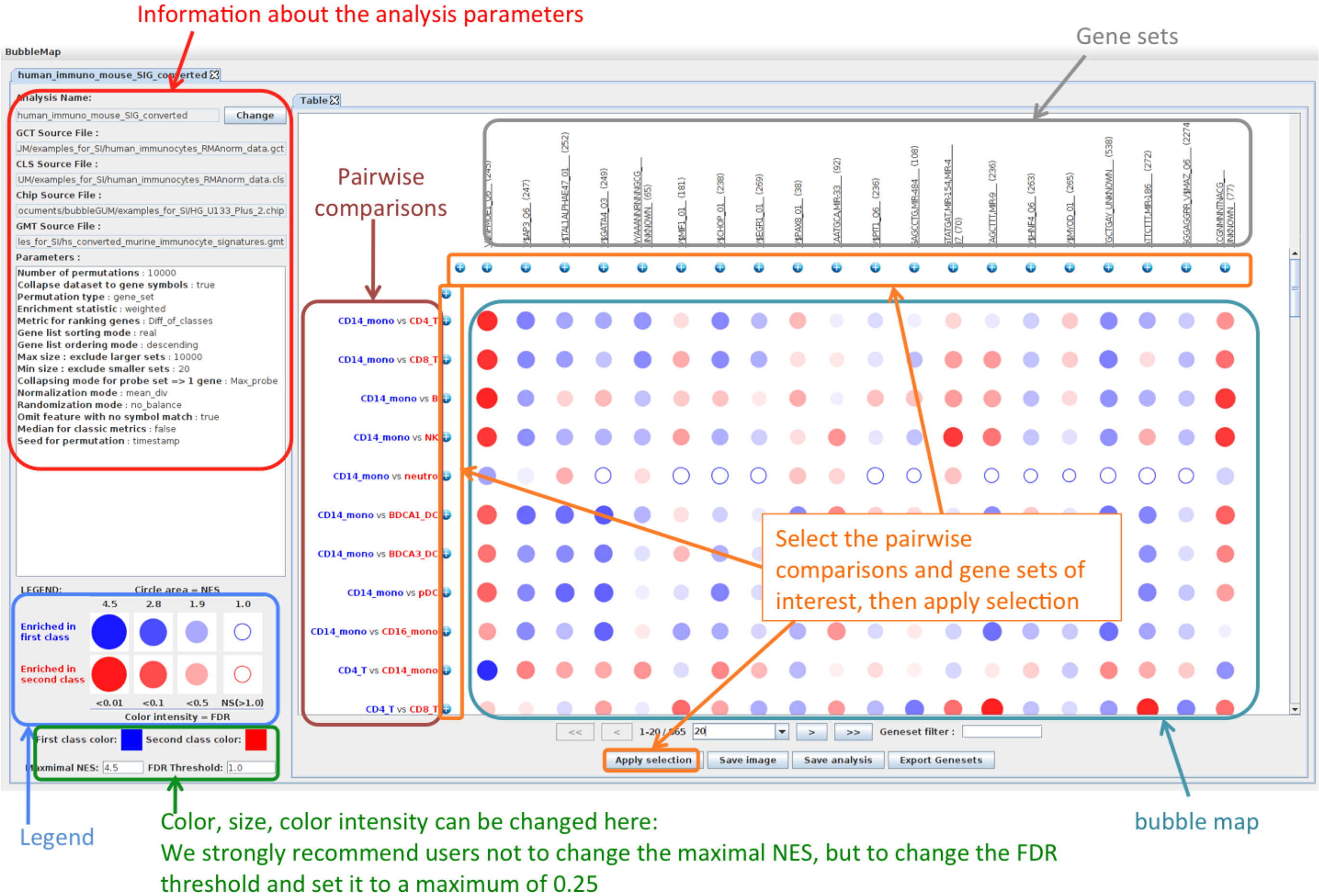

Figure 17: Raw output of BubbleMap. The upper left panel displays the analysis parameters. The bottom left panel displays the legend and the color/size tunable parameters. The right panel displays the bubble map, where each line represents a pairwise comparison and each column represents a gene set.

As mentioned in the manuscript, the output of BubbleMap can be optimized by filtering the gene sets and pairwise comparisons of interest, using the mouse to select them one by one, or using the Geneset filter "field" to look automatically for a gene set of interest (Figure 17). Then, applying the selection generates a new tab with the filtered bubble map. The outputs can be saved as high-definition (.svg) or rastered (.png) figures.

References

- Hochberg,Y. et al. (1990) More powerful procedures for multiple significance testing. Stat Med, 9(7):811-8.

- Bardou,P. et al. (2014) jvenn: an interactive Venn diagram viewer. BMC Bioinformatics 15:293 doi:10.1186/1471-2105-15-293

- Reiner,A. et al. (2003) Identifying differentially expressed genes using false discovery rate controlling procedures. Bioinformatics, 19(3):368-75.

- Subramanian,A. et al. (2005) Gene set enrichment analysis: a knowledge-based approach for interpreting genome-wide expression profiles. Proc Natl Acad Sci U S A, 102(43):15545-50.
