# Supplementary material for: BubbleGUM: automatic extraction of phenotype molecular signatures and comprehensive visualization of multiple Gene Set Enrichment Analyses
Source: BMC Genomics. 2015 Oct 19;16:814. doi: 10.1186/s12864-015-2012-4 (PMC4617899; doi:10.1186/s12864-015-2012-4)
Supplement: Additional file 3: Table S1. — Computational cost of BubbleMap. (DOCX 15 kb) [file 12864_2015_2012_MOESM3_ESM.docx]

**Additional 3: Table S1: Computational cost of BubbleMap.**

|  | Nb of gene sets^(2)^ | | | |
| --- | --- | --- | --- | --- |
|  | **50** | **100** | **500** | **1000** |
| 9 samples, 3 conditions^(1)^  (6 pairwise comparisons) | <20s**^(3)^**  <900 MB**^(4)^** | <1 min  <900 MB | 2 min  <900 MB | 4 min  <900 MB |
| 15 samples, 5 conditions  (20 pairwise comparisons) | <1 min  <1.2 GB | 2 min  <1.2 GB | 10 min  <1.2 GB | 20 min  <1.2 GB |
| 21 samples, 7 conditions  (42 pairwise comparisons) | 3 min  <1.4 GB | 5 min  <1.4 GB | 25 min  <1.4 GB | 50 min  <1.4 GB |

^(1)^Different datasets have been analyzed by BubbleMap using files composed of different gene set numbers; ^(2)^with a Desktop computer having a 2.4 GHz processor and 4 GB of RAM allocated. ^(3)^The approximate duration time and ^(4)^maximal RAM memory used for each job are indicated, for analyses with 1000 gene set based permutations and the Affymetrix Human Genome U133 Plus 2.0 gene chip baring over 50,000 probesets.
